# Diagnostic and prognostic biomarkers associated with histotype in advanced epithelial ovarian cancer

**DOI:** 10.1038/s41598-025-24938-0

**Published:** 2025-10-23

**Authors:** Ella Ittner, Hugo Swenson, Lucas Werner, Elisabeth Werner Rönnerman, Constantina Mateoiu, Anikó Kovács, Pernilla Dahm-Kähler, Ghassan Saed, Per Karlsson, Toshima Z. Parris, Khalil Helou

**Affiliations:** 1 Department of Oncology, Institute of Clinical Sciences, Sahlgrenska Academy, University of Gothenburg, Gothenburg, Sweden; 2 Sahlgrenska Center for Cancer Research, Sahlgrenska Academy, University of Gothenburg, Gothenburg, Sweden; 3 Region Västra Götaland, Department of Clinical Pathology, Sahlgrenska University Hospital, Gothenburg, Sweden; 4 Department of Obstetrics and Gynecology, Institute of Clinical Sciences, Sahlgrenska Academy, University of Gothenburg, Gothenburg, Sweden; 5 Department of Obstetrics and Gynecology, Wayne State University School of Medicine, Detroit, MI USA; 6 Department of Gynecologic Oncology, Karmanos Cancer Institute, Detroit, MI USA; 7 Department of Obstetrics and Gynecology, University of Jordan School of Medicine, Amman, Jordan

**Keywords:** Cancer, Computational biology and bioinformatics, Biomarkers, Molecular medicine, Oncology

## Abstract

Despite advances in cancer treatments, epithelial ovarian cancer (EOC) remains the leading cause of death among gynecologic cancers. EOC is stratified into five main histopathological subtypes: high-grade serous carcinoma (HGSC), low-grade serous carcinoma (LGSC), endometrioid carcinoma (EC), clear cell carcinoma (CCC), and mucinous carcinoma (MC). However, personalized treatment strategies and reliable biomarkers for all histotypes remain elusive. Building on our previous work with early-stage EOC, we aim to explore diagnostic and prognostic biomarkers in advanced-stage EOC, updated to the latest World Health Organization classification guidelines from 2020, using comprehensive transcriptomic profiling from total RNA sequencing of 146 EOCs. Differential expression analysis identified top 9 histotype-specific gene panels for HGSC, CCC, MC, and EC, including *S100A1* (HGSC), *ARID3A* (CCC), *LGALS4* (MC), and *PAX9* (EC). We also identified gene candidates associated with overall survival and disease-specific survival, reflecting both favorable (e.g., *OTOF*, *EEF1E1-BLOC1S5*, and *STAC3*) and unfavorable (e.g., *SMOC1*, *GDPGP1*, *EPRS1*) clinical outcome. Additionally, enrichment analysis revealed tumor progression-related pathways unique to each histotype, offering insights into the molecular mechanisms underlying disease progression and potential therapeutic targets. These findings provide valuable insights into the molecular landscape of advanced-stage EOC, paving the way for more effective diagnostic and prognostic tools across diverse histotypes.

**Supplementary Information:**

The online version contains supplementary material available at 10.1038/s41598-025-24938-0.

## Introduction

Ovarian cancer (OC) remains the deadliest of all gynecologic cancers, with an estimated 325,000 new cases and 207,000 deaths globally in 2022^1^. The overall 5-year survival rate is approximately 51% for early-stage disease (stage I and II), which drops to 31% for advanced-stage patients (stage III and IV)^2^. These survival outcomes are shaped by race, stage, histotype, and maximal cytoreductive surgery^3^. Biologically, ovarian cancer is a highly complex disease, originating from germ cells, stromal cells or predominantly epithelial cells, which account for 90% of OCs^4^.

Epithelial ovarian cancer (EOC) is considered a heterogeneous disease that is classified into five main histotypes: High-grade serous carcinoma (HGSC), low-grade serous carcinoma (LGSC), endometrioid carcinoma (EC), clear cell carcinoma (CCC), and mucinous carcinoma (MC). Each histotype is characterized by distinct clinical behaviors, incidence rates, molecular profiles, and proposed tissues of origin. Histotype-classification is often aided by immunohistochemistry (IHC) markers such as Wilms tumor protein (WT1) and p53 status for HGSC, Napsin A for CCC, and histotype-specific estrogen receptor (ER) and progesterone receptor (PR) expression^4–8^. HGSC, the most common histotype (~ 70%), likely originates in the fallopian tube and is associated with poor prognosis and frequent *TP53* and *BRCA* mutations^9^. LGSC (~ 3%), arises from ovarian or fallopian tube epithelium and is defined by indolent growth, *KRAS*/*BRAF* mutations, and chemoresistance. EC (~ 12%) and CCC (~ 12%) are often associated with endometriosis and display *PTEN*, *ARID1A*, and *PIK3CA* mutations, though EC tends to have a more favorable prognosis in early-stage disease compared to CCC. MC (~ 3%) resembles gastrointestinal tumors, and is characterized by *KRAS* mutations and poor therapeutic response^5,10,11^. Furthermore, several additional genes have been proposed to show histotype-specificity, such as the *MUC* family for MC, *HNF1B* and *ARID1A* for CCC, or *CTNNB1* for EC, complementing clinically applied markers^12–15^.

Current treatment strategies for OC do not account for the distinct biological and clinical differences among the histotypes, thereby limiting therapy to maximal cytoreductive surgery followed by platinum-based chemotherapy^16^. Though promising in other cancer types, immunotherapy has only shown modest results in EOC due to its suppressive tumor microenvironment^17^. Notably, therapeutic improvements include the application of poly ADP ribose polymerase inhibitors (PARPi) for *BRCA* mutation carriers and hyperthermic intraperitoneal chemotherapy (HIPEC) for advanced-stage disease^18,19^. However, these treatments are not widely effective across all histotypes, underscoring the need for personalized approaches.

Apart from the established protein-based tools used for histotype stratification, comprehensive molecular biomarkers - including genomic, transcriptomic, and circulating markers - for early detection, prognosis, or therapeutic guidance remain limited in clinical application for EOC. The general tumor marker carbohydrate antigen 125 (CA125), the most commonly used tumor marker in EOC, lacks sensitivity and specificity, particularly in early-stage disease^20^. Efforts to develop multi-marker panels and risk assessment algorithms have shown potential, with human epididymis secretory protein 4 (HE4) emerging as a promising candidate due to its high specificity for ovarian epithelial tissue, albeit with low sensitivity^21,22^.

While HGSC has been the primary focus of research and treatment innovations, other histotypes remain understudied, creating a gap in our understanding of histotype-specific biology and therapeutic responses. Building on our prior work on early-stage EOC^23–25^, this study explores histotype-specific biomarkers in a cohort comprised of advanced-stage EOC reclassified based on all five major histotypes, along with benign and borderline cases. The primary focus was on HGSC, EC, MC, and CCC, aiming to identify comprehensive molecular gene panels that stratify the histotypes and prognostic markers using long-term clinical follow-up, thereby complementing histotype-based classification and supporting the development of tailored therapeutic approaches. This will ultimately enhance outcomes for ovarian cancer patients.

## Results

### Retrospective reclassification according to the recent WHO guidelines highlights its importance for long-term EOC analysis

**Fig. 1:**
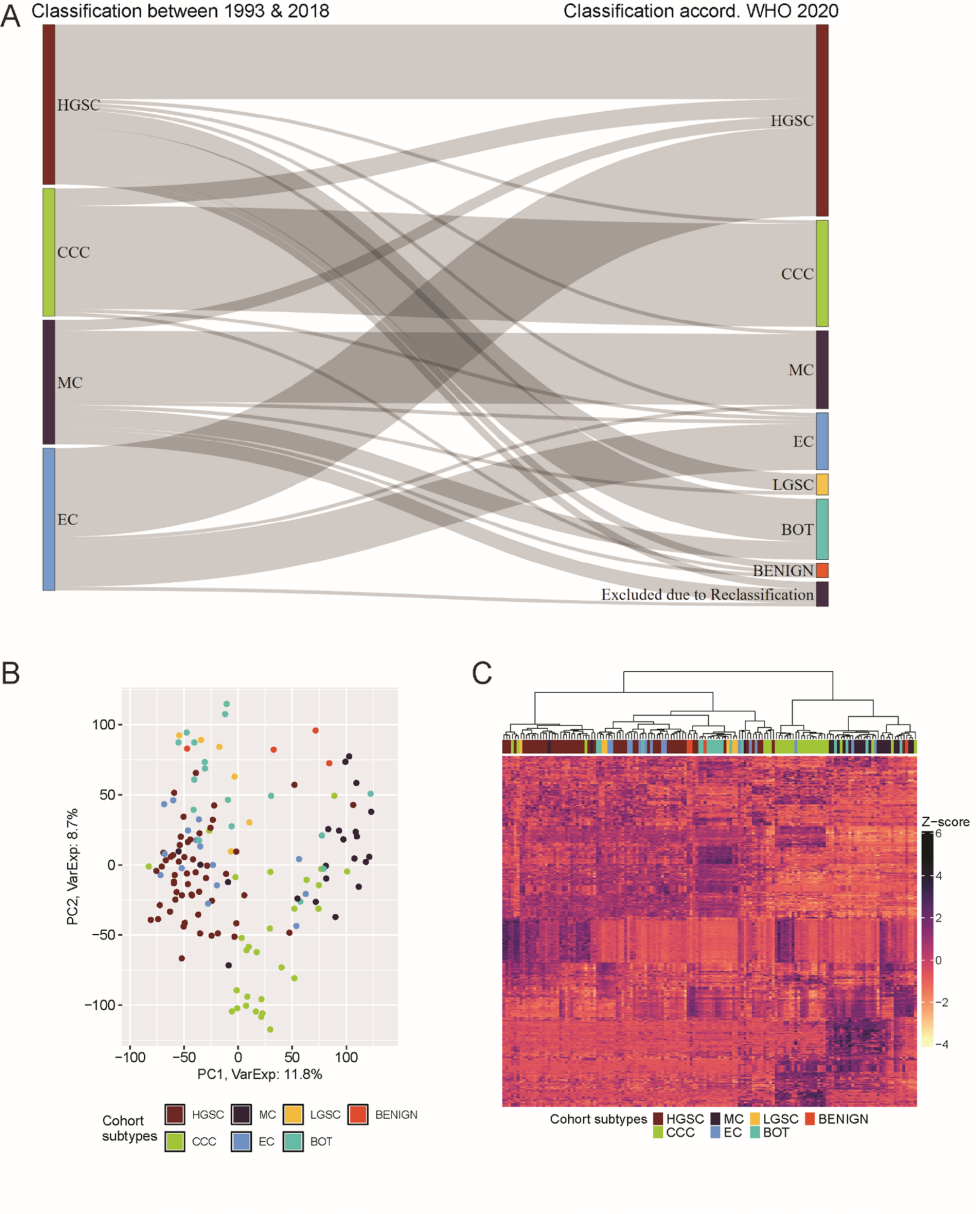
Transcriptional landscape and genetic variance in the advanced-stage EOC study cohort reclassified according to the 2020 WHO guidelines. (**A**) Sankey diagram visualizing the impact of histotype reclassification based on pathology (47% of cases reclassified according to WHO 2020). (**B**) PCA plot illustrating histotype-stratified clustering within the cohort. PCA was performed on variance-stabilized expression values from all annotated Ensembl gene IDs, as processed by DESeq2. (**C**) Heatmap with hierarchical clustering (Euclidian distance, Ward.D2 clustering criterion) of the top 500 most variable genes, stratified by cohort subtypes. Abbreviations: BOT: borderline tumor, CCC: clear cell carcinoma, EC: endometrioid carcinoma, HGSC: high-grade serous carcinoma, LGSC: low-grade serous carcinoma, MC: mucinous carcinoma, PCA: principal component analysis, WHO: World Health Organization.

After reclassification of the histotypes in accordance with the updated WHO guidelines, 73 of the 156 (47%) malignant EOC samples were reclassified into a different histopathological subtype (Supplementary Table 1). The original cohort composition included approximately 29% HGSC, 23% CCC, 26% EC, and 22% MC. Reclassification introduced additional histotypes, such as 6 cases of low-grade serous carcinoma (LGSC) and 21 non-malignant cases, comprising 17 borderline tumors (BOT) and 4 benign cases. Notably, 12 HGSCs and 5 MCs were reclassified as BOT, now collectively grouped under the term “BOT”. Two samples could not be reclassified due to unavailable FFPE material, while five were excluded from further analysis after being reclassified into malignant categories outside the scope of malignant EOC. The final cohort distribution after reclassification included 35% HGSC, 19% CCC, 14% MC, 11% BOT, 10% EC, 4% LGSC, 3% benign, and 4% excluded (Fig. 1A). This underscores the importance of updating sample classification according to the current guidelines in long-term cancer studies to maintain data comparability and relevance to contemporary diagnostic and therapeutic strategies.

### Cohort characteristics and survival outcomes

The final cohort was predominantly comprised of advanced-stage tumors, with 84% being stage III and 13% stage IV (Supplementary Table 2). The median age at diagnosis was 54 years (interquartile range [IQR]: 45.00, 65.25). Among malignant cases, the average survival time was 5.4 years and 84% of patients succumbed to their disease. Survival rates varied significantly by histotype. Among the malignant cohort, 37% of patients survived beyond five years. MC had the lowest 5-year survival rates (23%), followed by 31% for EC, 37% for CCC, 41% for HGSC, and 67% for LGSC. All histotypes were associated with a higher risk of death due to EOC than other causes (Supplementary Fig. 1). These differences emphasize the varying prognoses among EOC histotypes, with MC showing the most unfavorable prognosis, while LGSC demonstrated comparatively better outcomes.

### Transcriptomic landscape of advanced-stage EOC

To explore the global transcriptomic landscape of EOC, principal component analysis (PCA) was performed on RNA sequencing (RNA-seq) expression profiles derived from 46,122 Ensembl-annotated gene identifiers (Fig. 1B). PCA revealed both overlapping and distinct expression profiles across the seven cohort subtypes. Clear separation of the clusters was observed for CCC and MC, reflecting their distinct transcriptomic signatures. HGSC largely clustered with LGSC and EC, indicating shared expression profiles among these subtypes. Notably, a subset of EC samples overlapped with the CCC and MC clusters, suggesting transcriptional heterogeneity within the EC group. BOT samples showed variable positioning, with some aligning with benign profiles and others, particularly mucinous BOTs, clustering near MC. These patterns underscore the molecular complexity and inter-subtype transitions within EOC. The heatmap of the 500 most variable genes reinforced these trends, with CCC forming the most distinct cluster, followed by MC and BOT (Fig. 1C). HGSC intermingled with LGSC, EC, and BOT, while EC exhibited notable heterogeneity. These findings highlight the extensive transcriptomic overlap in advanced-stage EOC while emphasizing distinct expression profiles in CCC and MC. The observed heterogeneity within EC and overlapping clusters across subtypes further underscore the complexity of EOC’s molecular landscape, warranting deeper investigation.

### Differential gene expression and identification of histotype-specific biomarker panels

**Fig. 2:**
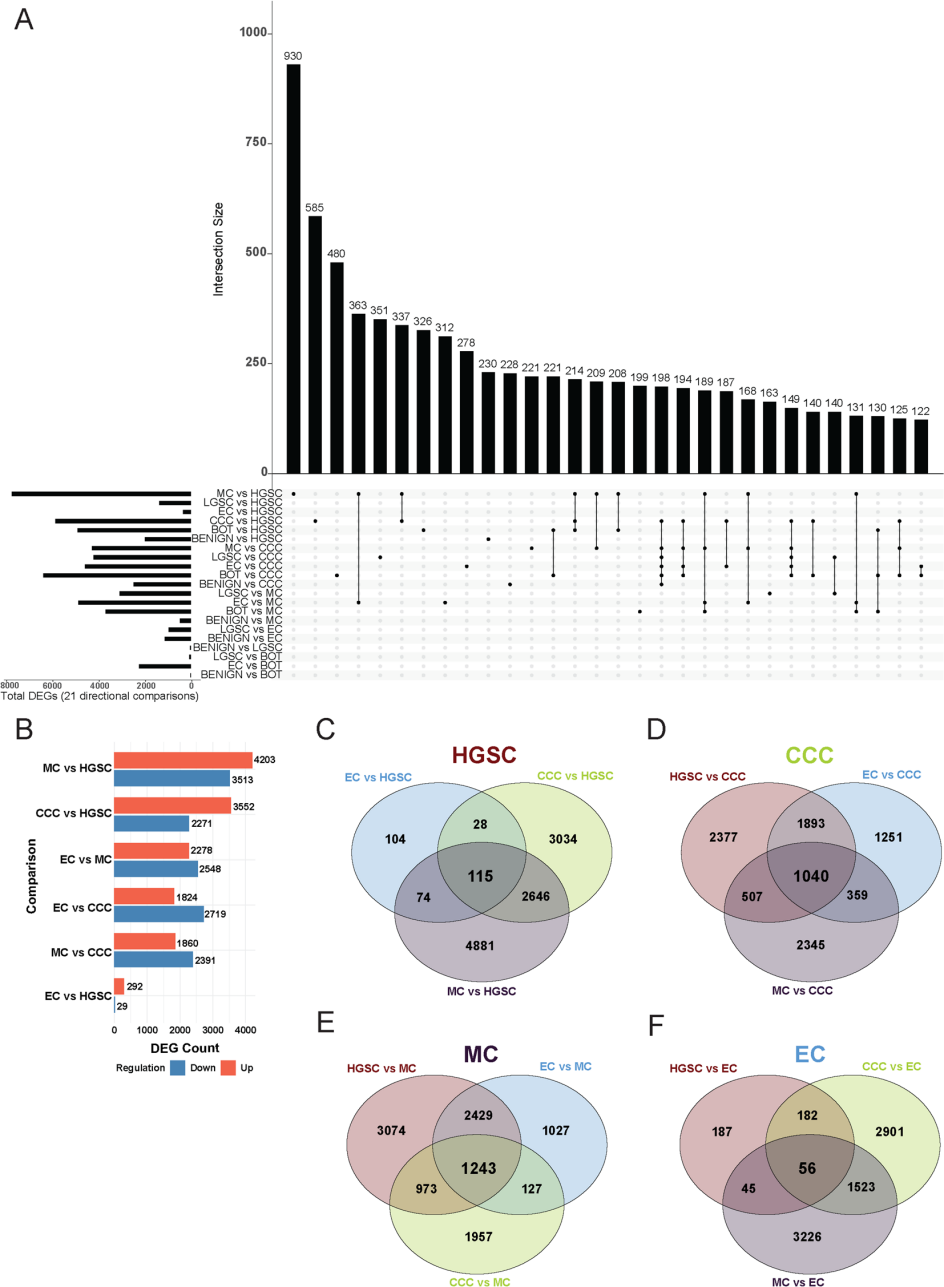
DEG comparisons and the identification of histotype-specific biomarkers in advanced-stage EOC. (**A**) UpSet plot illustrating the overlap of DEGs across 21 directional histotype comparisons, each defined by selecting the histotype with the larger sample size as reference. Left-side bars indicate the total number of DEGs per comparison, while top bars (Intersection Size) represent the number of DEGs shared across one or more comparisons. Intersections are ordered by decreasing frequency, revealing that most DEGs are unique to individual comparisons, with limited transcriptomic overlap between histotypes. (**B**) Bar plot showing the number of upregulated and downregulated DEGs for six directional comparisons involving the four main malignant histotypes (HGSC, CCC, MC, and EC). DEG counts ranged from 321 (EC vs. HGSC) to 7,716 (MC vs. HGSC). The proportion of upregulated DEGs ranged from 40% (EC vs. CCC) to 91% (EC vs. HGSC), with most contrasts exhibiting a relatively balanced distribution (~ 40–60%). (**C-F**) Venn diagrams showing DEG overlap and histotype-specific candidate biomarkers for (C) HGSC, (**D**) CCC, (E) MC, and (**F**) EC. These gene sets represent subtype-enriched transcriptomic signatures with potential diagnostic and stratification value in advanced-stage EOC. Abbreviations: BOT: borderline tumor, CCC: clear cell carcinoma, DEG: differentially expressed gene/s, EC: endometrioid carcinoma, HGSC: high-grade serous carcinoma, LGSC: low-grade serous carcinoma, MC: mucinous carcinoma.

A comprehensive differential gene expression (DGE) analysis was performed to systematically identify differentially expressed genes (DEGs) between histotypes across 21 directional pairwise comparisons (Supplementary Data 1). In total, 18,590 transcripts were differentially expressed (p-adj < 0.05; |log₂ fold change| ≥ 1.0) in at least one comparison. The number of DEGs per comparison ranged from 2 (benign vs. BOT) to 7,719 (MC vs. HGSC), with a median of 2,578 DEGs per comparison (Fig. 2A). Comparisons involving benign cases yielded substantially fewer DEGs and showed limited overlap with malignant subtypes, reflecting broad transcriptomic divergence. Recurrent DEGs varied widely: 6,734 genes were differentially expressed in only one comparison, while 138 were found in ≥ 10 comparisons. No single DEG was consistently shared across all directional 21 comparisons, reflecting both histotype-specific expression patterns and shared transcriptional alterations across subsets of EOC.

To better capture consistent expression patterns, we focused on genes differentially expressed in all comparisons involving a given histotype (HGSC, CCC, MC, and EC), based on the analytic subset described in Supplementary Table 2. The number of DEGs ranged from 7,716 (MC vs. HGSC, with 4,203 upregulated and 3,513 downregulated) to 312 (EC vs. HGSC, with 292 upregulated and 29 downregulated; Fig. 2B), with several overlapping between subtypes (Fig. 2C-F). In total, 56 candidate biomarkers were identified for EC and 115 for HGSC. MC exhibited 1,040 histotype-specific genes, reflecting distinct clustering, while CCC was characterized by 1,243 histotype-specific genes, indicating strong differentiation from the other histotypes.

### Identification and validation of histotype-specific gene panels for advanced-stage EOC

To identify robust gene panels for advanced-stage EOC, we performed rigorous internal validation of the histotype-specific biomarker candidates. Logistic regression models with 10-fold cross-validation assessed ROC AUC, sensitivity, and specificity for each DEG. A random forest model computed a Global Importance Score for each gene. Gene filtering was based on a ROC AUC threshold of 0.7. Totally, 964 genes (77%) passed the threshold for MC, with a mean ROC AUC of 0.779 ± 0.046 and an average Importance score of 0.036 ± 0.065. For CCC, 695 genes (67%) met the criteria, with a mean ROC AUC of 0.78 ± 0.052 and an Importance score of 0.058 ± 0.115. HGSC had 57 validated genes (50%), with a mean ROC AUC of 0.752 ± 0.039 and an Importance score of 0.897 ± 0.699. Only 10 genes (17%) passed the threshold for EC, with a mean ROC AUC of 0.741 ± 0.032 and an Importance score of 0.585 ± 0.265. These results reveal histotype-specific differences, with MC and CCC showing stronger predictive values for advanced-stage EOC.

External validation was performed using RNA expression datasets (GSE2109, GSE6008, GSE44104, EMTAB1814), focusing on genes with annotated Hugo Gene Nomenclature Committee (HGNC) symbols. Of the DEGs, 744 of 1,040 CCC genes had valid HGNC symbols, with 24 externally validated (2.3%). MC had 895 of 1,246 valid genes, with 11 validated (0.9%). EC had 48 of 58 valid genes but no external validation (0%). HGSC had 102 of 115 valid genes, with 10 validated (8.7%). Given the limited validation across histotypes, external datasets primarily aided in annotating final protein-coding hits, offering insights for validated genes.

**Table 1:** Summary of histotype-specific transcriptomic biomarker candidates for advanced-stage EOC. This table lists histotype-specific biomarker candidates identified for the four main histotypes of advanced-stage EOC (CCC, MC, EC, HGSC). The gene panels represent histotype-specific transcriptomic signatures with potential diagnostic or therapeutic relevance, as well as prognostic candidates for overall survival and disease-specific survival where available. For each gene, the table provides gene type (Ensembl annotation), ensembl ID, regulation pattern (up/down in the respective histotype versus all others), predictive performance metrics (internal validation: ROC-AUC, C-index, Sensitivity/Specificity, importance Score), and external validation status as well as proteomic concordance status. Biomarkers are grouped by histotype to facilitate direct comparison. Abbreviations: CCC, clear cell ovarian carcinoma; DEG, differentially expressed gene; EC, endometrioid ovarian carcinoma; HGSC, High-grade serous ovarian carcinoma; MC, mucinous ovarian carcinoma; ROC-AUC, receiver operating characteristic area under the curve; Sens, Sensitivity; Spec, Specificity; C-index, concordance index; CI, confidence interval; HR, hazard ratio; IS, importance score; DSS, Disease-specific survival; OS, overall survival.

Histotype	Transcriptomic biomarker classification		Biomarker candidate	Gene type (Ensembl annotation)	Ensembl ID		Regulation		Predictive performance		External validation status		
											External transcriptomic validation		Proteomic concordance
*HGSC*	Histotype-specific gene panel		HNF1A	protein coding	ENSG00000135100	Up		ROC AUC = 0.86; Sens = 0.71; Spec = 0.82; IS = 3.24		no			
			COL17A1	protein coding	ENSG00000065618	Up		ROC AUC = 0.86; Sens = 0.81; Spec = 0.74; IS = 2.73		no		validated on protein level (2/3)	
			S100A1	protein coding	ENSG00000160678	Down		ROC AUC = 0.8; Sens = 0.72; Spec = 0.81; IS = 2.64		no		validated on protein level (2/3)	
			ENSG00000255085	transcribed unprocessed pseudogene	ENSG00000255085	Down		ROC AUC = 0.83; Sens = 0.74; Spec = 0.81; IS = 2.59		no		-	
			NPM2	protein coding	ENSG00000158806	Up		ROC AUC = 0.82; Sens = 0.8; Spec = 0.76; IS = 1.99		no			
			ARSL	protein coding	ENSG00000157399	Up		ROC AUC = 0.79; Sens = 0.94; Spec = 0.62; IS = 1.73		no		validated on protein level (2/3)	
			RPL7AP75	transcribed processed pseudogene	ENSG00000259674	Up		ROC AUC = 0.82; Sens = 0.85; Spec = 0.68; IS = 1.6		no		-	
			RPL23P6	processed pseudogene	ENSG00000180211	Up		ROC AUC = 0.75; Sens = 0.9; Spec = 0.54; IS = 1.59		no		-	
			OR5BA1P	transcribed unprocessed pseudogene	ENSG00000255303	Up		ROC AUC = 0.76; Sens = 0.86; Spec = 0.58; IS = 1.42		no			
	Prognostic biomarker candidates	OS	STAC3	protein coding	ENSG00000185482	-		C-index = 0.70; HR = 0.33; CI [0.18–0.61.18.61]; p.adj = 0.001		yes			
			EPRS1	protein coding	ENSG00000136628	-		C-index = 0.74; HR = 2.26; CI [1.51–3.36.51.36]; p.adj < 0.001		-			
		DSS	ENSG00000263220	lncRNA	ENSG00000263220	-		C-index = 0.75; HR = 0.29; CI [0.15–0.54.15.54]; p.adj < 0.001		-		-	
			BARX1	protein coding	ENSG00000131668	-		C-index = 0.69; HR = 2.46; CI [1.40–4.32.40.32]; p.adj = 0.004		yes			
*CCC*	Histotype-specific gene panel		ENSG00000287125	lncRNA	ENSG00000287125	Down		ROC AUC = 0.9; Sens = 0.71; Spec = 0.98; IS = 0.9		no		-	
			C16orf74	protein coding	ENSG00000154102	Down		ROC AUC = 0.9; Sens = 0.71; Spec = 0.96; IS = 0.8		no			
			ENSG00000229853	processed pseudogene	ENSG00000229853	Down		ROC AUC = 0.95; Sens = 0.86; Spec = 0.94; IS = 0.76		no		-	
			ATP11A-AS1	lncRNA	ENSG00000232684	Down		ROC AUC = 0.9; Sens = 0.79; Spec = 0.92; IS = 0.67		no		-	
			ATP11A	protein coding	ENSG00000068650	Down		ROC AUC = 0.9; Sens = 0.79; Spec = 0.94; IS = 0.67		no		validated on protein level; histotype-specifc (3/3)	
			GGT6	protein coding	ENSG00000167741	Up		ROC AUC = 0.89; Sens = 0.82; Spec = 0.93; IS = 0.57		no			
			ARID3A	protein coding	ENSG00000116017	Down		ROC AUC = 0.89; Sens = 0.84; Spec = 0.86; IS = 0.42		yes		validated on protein level; histotype-specifc (3/3)	
			PDE11A	protein coding	ENSG00000128655	Up		ROC AUC = 0.9; Sens = 0.79; Spec = 0.84; IS = 0.4		no			
			KSR1	protein coding	ENSG00000141068	Down		ROC AUC = 0.9; Sens = 0.92; Spec = 0.66; IS = 0.4		no		validated on protein level (2/3)	
	Prognostic biomarker candidates	OS	OTOF	protein coding	ENSG00000115155	-		C-index = 0.83; HR = 0.12; CI [0.03–0.46.03.46]; p.adj = 0.006		-		-	
			SMOC1	protein coding	ENSG00000198732	-		C-index = 0.83; HR = 8.75; CI [2.55–30.08.55.08]; p.adj = 0.002		-		-	
		DSS	ENSG00000270426	lncRNA	ENSG00000270426	-		C-index = 0.90; HR = 0.13; CI [0.04–0.50.04.50]; p.adj = 0.011		-		-	
			ENSG00000264272	lncRNA	ENSG00000264272	-		C-index = 0.86; HR = 9.61; CI [2.13–43.34.13.34]; p.adj = 0.013		-		-	
*MC*	Histotype-specific gene panel		CREB3L1	protein coding	ENSG00000157613	Down		ROC AUC = 0.91; Sens = 0.86; Spec = 0.93; IS = 0.73		no			
			ENSG00000248515	lncRNA	ENSG00000248515	Up		ROC AUC = 0.93; Sens = 0.86; Spec = 0.94; IS = 0.67		no		-	
			LINC02438	lncRNA	ENSG00000248238	Up		ROC AUC = 0.92; Sens = 0.91; Spec = 0.87; IS = 0.65		no		-	
			RPL21P46	processed pseudogene	ENSG00000241612	Up		ROC AUC = 0.92; Sens = 0.86; Spec = 0.9; IS = 0.5		no		-	
			LINC00958	lncRNA	ENSG00000251381	Up		ROC AUC = 0.89; Sens = 0.86; Spec = 0.92; IS = 0.43		no		-	
			ERN2	protein coding	ENSG00000134398	Down		ROC AUC = 0.88; Sens = 0.81; Spec = 0.89; IS = 0.39		no			
			LGALS4	protein coding	ENSG00000171747	Down		ROC AUC = 0.91; Sens = 0.86; Spec = 0.87; IS = 0.35		no		validated on protein level; histotype-specifc (3/3)	
			CELSR2	protein coding	ENSG00000143126	Up		ROC AUC = 0.92; Sens = 0.86; Spec = 0.88; IS = 0.32		no			
			ENSG00000249441	lncRNA	ENSG00000249441	Up		ROC AUC = 0.95; Sens = 0.91; Spec = 0.92; IS = 0.29		no		-	
	Prognostic biomarker candidates	OS	ENSG00000201483	protein coding	ENSG00000201483	-		C-index = 0.91; HR = 0.12; CI [0.02–0.70.02.70]; p.adj = 0.028		-			
			ENSG00000278215	protein coding	ENSG00000278215	-		C-index = 0.92; HR = 7.25; CI [1.52–34.43.52.43]; p.adj = 0.019		-			
		DSS	ENSG00000201483	lncRNA	ENSG00000201483	-		C-index = 0.91; HR = 0.12; CI [0.02–0.70.02.70]; p.adj = 0.028		-		-	
			ENSG00000278215	lncRNA	ENSG00000278215	-		C-index = 0.92; HR = 7.25; CI [1.52–34.43.52.43]; p.adj = 0.019		-		-	
*EC*	Histotype-specific gene panel		KCNF1	protein coding	ENSG00000162975	Down		ROC AUC = 0.69; Sens = 0.67; Spec = 0.67; IS = 1.07		no			
			CPLX2	protein coding	ENSG00000145920	Down		ROC AUC = 0.73; Sens = 0.67; Spec = 0.78; IS = 1.05		no			
			LINC03053	lncRNA	ENSG00000223486	Down		ROC AUC = 0.78; Sens = 0.79; Spec = 0.63; IS = 0.9		no		-	
			ENSG00000267749	lncRNA	ENSG00000267749	Up		ROC AUC = 0.81; Sens = 0.67; Spec = 0.78; IS = 0.76		no		-	
			NRIP3-DT	lncRNA	ENSG00000253973	Down		ROC AUC = 0.65; Sens = 0.79; Spec = 0.46; IS = 0.74		no		-	
			PLA2G4A	protein coding	ENSG00000116711	Down		ROC AUC = 0.68; Sens = 0.53; Spec = 0.84; IS = 0.72		no			
			EYA1	protein coding	ENSG00000104313	Down		ROC AUC = 0.71; Sens = 0.6; Spec = 0.85; IS = 0.63		no			
			PAX9	protein coding	ENSG00000198807	Down		ROC AUC = 0.73; Sens = 0.53; Spec = 0.81; IS = 0.58		no			
			SCUBE2	protein coding	ENSG00000175356	Down		ROC AUC = 0.64; Sens = 0.67; Spec = 0.59; IS = 0.57		no			
	Prognostic biomarker candidates	OS	EEF1E1-BLOC1S5	protein coding	ENSG00000265818	-		C-index = 0.92; HR = 0.11; CI [0.03–0.47.03.47]; p.adj = 0.004		-			
			GDPGP1	protein coding	ENSG00000183208	-		C-index = 0.93; HR = 9.63; CI [1.72–53.83.72.83]; p.adj = 0.030		-			

**Fig. 3:**
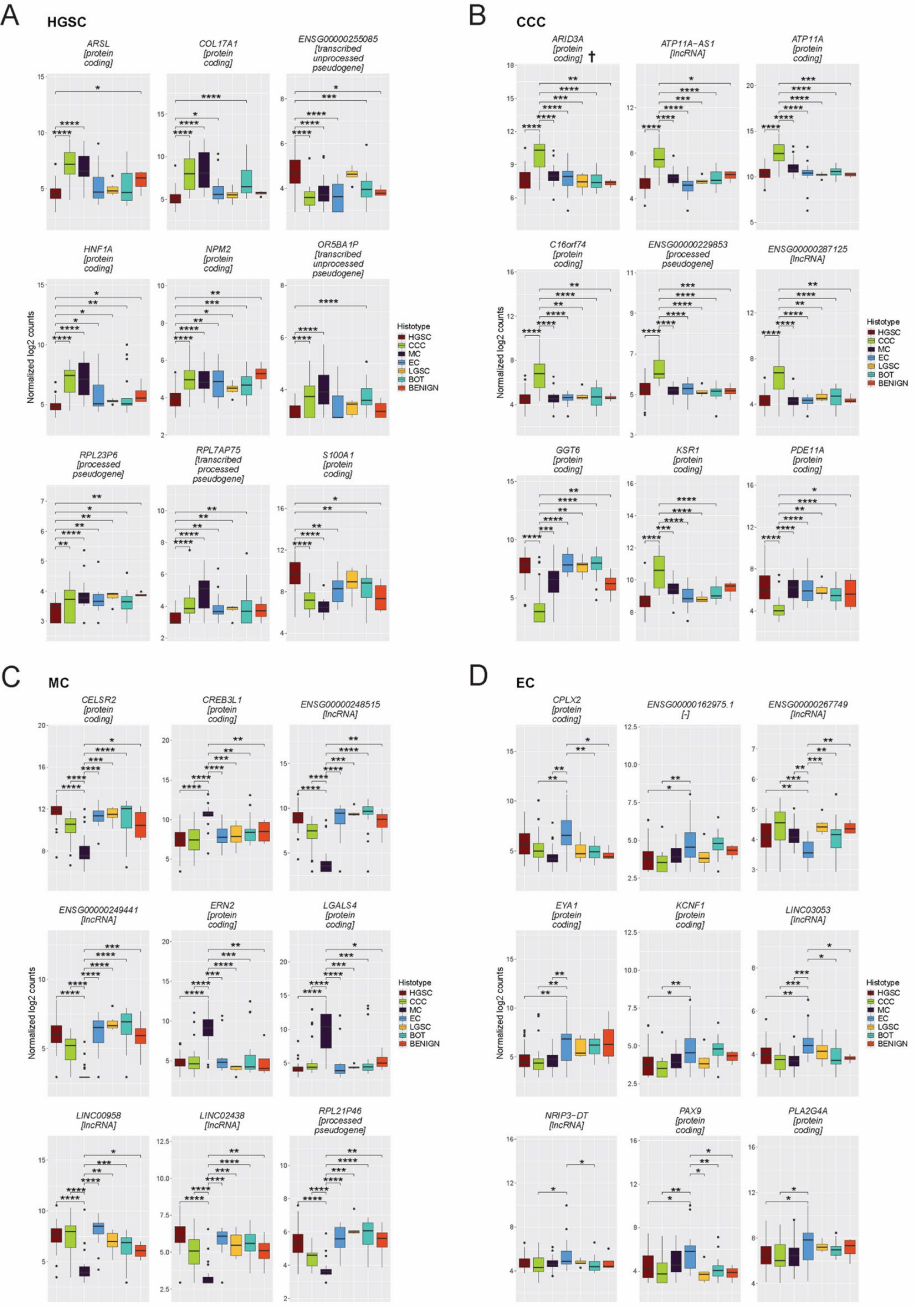
Expression of histotype-specific gene panels in advanced-stage EOC. Boxplots displaying normalized log2 expression counts for the top nine genes identified for each histotype, selected based on the highest ROC AUC and importance scores. These genes, encompassing both protein-coding genes and non-coding RNAs, were selected from the final histotype-specific gene panels for (**A**) HGSC, (**B**) CCC, (**C**) MC, and (**D**) EC. The boxplots illustrate differential expression patterns across seven cohort tumor subtypes, emphasizing the biological relevance and predictive potential of these genes as histotype-specific biomarkers. Pairwise comparisons were performed using the Wilcoxon test with Benjamini-Hochberg (BH) FDR correction. Statistical significance is indicated by asterisks: *p < 0.05, **p < 0.01, ***p < 0.001, and ****p < 0.0001. These comparisons highlight the robustness of the markers in distinguishing histotypes, supporting their application as diagnostic tools in advanced-stage EOC. Abbreviations: CCC: clear cell carcinoma, DEG: differentially expressed genes, EC: endometrioid carcinoma, EOC: epithelial ovarian cancer, FDR: False-discovery rate, HGSC: high-grade serous carcinoma, LGSC: low-grade serous carcinoma, MC: mucinous carcinoma.

Final gene panels for advanced-stage EOC were constructed by selecting the top 25 genes with the highest ROC AUC, followed by the top 9 genes with the highest Importance scores. These panels not only contain protein-coding genes, but also non-coding RNAs and pseudogenes, reflecting the diverse molecular landscape of advanced EOC (Table 1; Fig. 3, Supplementary Data 2). The additional cohort subtypes (LGSC, BOT, benign) were included in expression plots for visualization only and were not used in panel selection. The final histotype-specific biomarker panels for each four main histotypes were (a) HGSC (*ARSL*,* COL17A1*,* ENSG00000255085*,* HNF1A*,* NPM2*,* OR5BA1P RPL7AP75*,* RPL23P6*,* S100A1*), (b) CCC (*ARID3A*,* ATP11A*,* ATP11A-AS1*,* C16orf74*,* ENSG00000229853*,* GGT6*,* KSR1*,* PDE11A*), (c) MC (*CELSR2*, *CREB3L1*,* ENSG00000248515*,* ENSG00000249441*,* ERN2*,* LGALS4*,* LINC00958*,* LINC02438*,* RPL21P46*), and (d) EC (*CPLX2*,* ENSG00000267749*,* EYA1*,* KCNF1*,* LINC03053*,* NRIP3-DT*,* PAX9*,* PLA2G4A*,* SCUBE2*).

Of the top nine genes for HGSC (Fig. 3A), seven candidate biomarkers (*HNF1A*,* COL17A1*,* NPM2*,* ARSL*,* RPL7AP75*,* RPL23P6*, and *OR5BA1P)* were underexpressed and two genes (*S100A1* and transcribed pseudogene *ENSG00000255085)* were overexpressed compared with the other three main histotypes as well as LGSC, benign tumors, and BOT. In contrast, CCC (Fig. 3B) showed a distinct pattern of overexpression, with seven candidates (*ENSG00000287125*,* C16orf74*,* ENSG00000229853*,* ATP11A-AS1*,* ATP11A*,* ARID3A*,* KSR1*) showing strong upregulation, while two candidates (*GGT6* and *PDE11A*) were downregulated, displaying significant differential expression across all pairwise comparisons. The MC biomarker panel (Fig. 3C) exhibited similar expression profiles to the other cohort subgroups, comparable to the HGSC candidates, with six genes (*ENSG00000248515*,* LINC02438*,* RPL21P46*,* LINC00958*,* CELSR2*,* ENSG00000249441*) showing reduced expression while *CREB3L1*,* ERN2* and *LGALS4* showed significantly elevated expression across the whole cohort. EC (Fig. 3D) exhibited the weakest differentiation, with most candidates underexpressed compared with other histotypes and limited statistical significance in the pairwise comparisons, highlighting challenges in identifying robust EC-specific biomarkers. The MC and CCC biomarker panels demonstrated exceptional robustness, with statistically significant differences in all pairwise comparisons. These findings support their potential utility as diagnostic tools for advanced-stage EOC. Lastly, to assess whether the transcriptomic-based histotype signals persist on the translational (protein) level, an integrative analysis of RNA and protein expression using a nearly paired proteomic-based advanced-stage EOC cohort was performed. Of the 36 histotype-specific DEGs in the panels, 21 were protein-coding and therefore eligible for protein assessment (Table 1); 12 of these were detected in the proteomics dataset. Using the same direction-and-significance consistency logic as in the performed external validation, which required concordant effect direction and statistical significance across at least two of the three histotype contrasts (e.g., HGSC vs. CCC, HGSC vs. EC, HGSC vs. MC), we classified seven candidates (*ARID3A*, *ARSL*, *ATP11A*, *COL17A1*, *KSR1*, *LGALS4*, *S100A1*) as validated on the protein level. Three of the candidates (HGSC: *COL17A1*, *ARSL*, *S100A1*; CCC: *KSR1*) were identified in two of three comparisons, while three genes were concordant in all three comparisons (CCC: *ATP11A* and *ARID3A*; MC: *LGALS4*). In addition, *PLA2G4A* and *SCUBE2* (EC) and *CELSR2* (MC) were not statistically significant on the protein-level, but showed consistent direction in at least two of three comparisons, supporting the cross-omic stability of these signals (Supplementary data 7).

### Histotype-stratified enrichment analysis reveals shared and unique molecular pathways in advanced-stage EOC

**Fig. 4:**
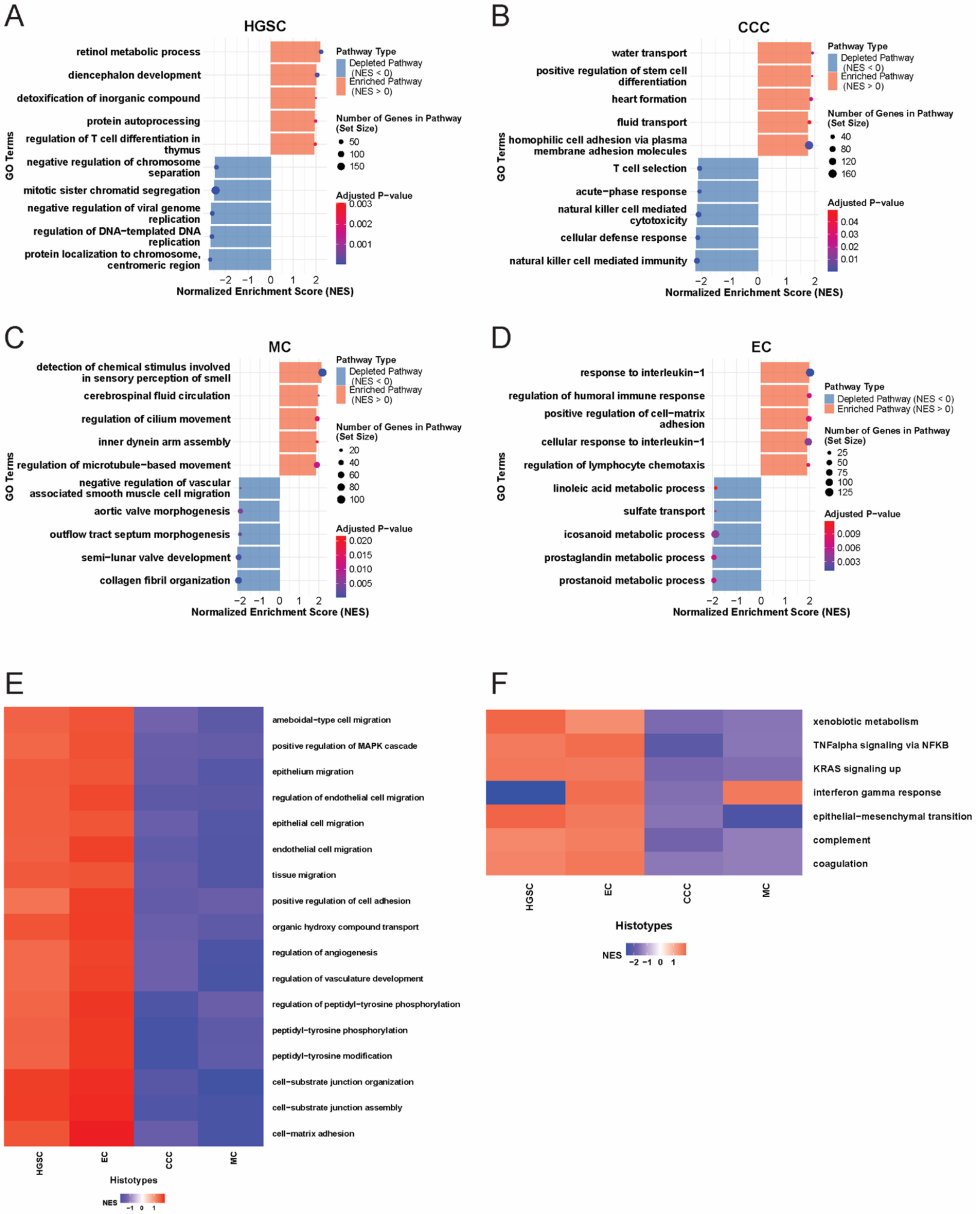
Comprehensive histotype-specific pathway analysis in advanced-stage EOC. Gene Set Enrichment Analysis (GSEA) was performed on differentially expressed genes (DEGs), identified using DESeq2, comparing each histotype individually against all others, identified unique biological pathways enriched in each histotype (**A-D**) to reveal distinct molecular interactions and shared pathways (**E**) enriched across multiple histotypes, highlighting overlapping mechanisms of tumorigenesis. (**F**) Hallmark pathway analysis of the DEGs using the same approach, identified the most significantly enriched cancer-related pathways. These findings capture both general tumorigenesis mechanisms and histotype-specific pathway activation emphasizing the diverse molecular landscape of advanced-stage EOC. Abbreviations: DEG: differentially expressed genes, EOC: epithelial ovarian cancer.

For the four main histotypes (HGSC, MC, EC, and CCC), histotype-stratified gene set enrichment analysis (GSEA) provided insights into unique and shared molecular signatures in advanced-stage EOC (Supplementary Data 3). A total of 1408 significantly enriched GO terms (p-value < 0.05; absolute Normalized Enrichment Score abs(NES) > 1) were identified, with 950 unique to a single histotype, 362 shared by two histotypes, 79 shared by three histotypes, and 17 enriched across all histotypes. HGSC exhibited the highest number of unique pathways (*n* = 498), followed by CCC (*n* = 225), MC (*n* = 168), and EC (*n* = 59). Statistical analysis of NES values revealed distinct enrichment trends: MC showed an overall negative enrichment, HGSC exhibited mixed enrichment and depletion, EC had moderate positive enrichment, and CCC showed predominantly negative enrichment. GSEA identified histotype-specific molecular signatures, with pathways showing enrichment (NES > 0) or depletion (NES < 0; Fig. 4A-D). HGSC (Fig. 4A) exhibited enrichment in metabolic and mitotic regulation pathways including “Retinol Metabolic Process”, “Detoxification of Inorganic Compounds”, and “Negative Regulation of Chromosome Separation”. In contrast, CCC (Fig. 4B) showed enrichment in pathways related to water transport and stem cell differentiation, while immune-related pathways, such as “Natural Killer Cell-Mediated Cytotoxicity” were suppressed. MC (Fig. 4C) showed enrichment in pathway regulation of cilium movement and depletion in collagen fibril organization, suggesting microtubule regulation and extracellular matrix (ECM) remodeling. EC (Fig. 4D) was enriched in immune response pathways, like “response to Interleukin-1” and regulation of humoral immune response, with suppression of metabolic pathways such as prostaglandin metabolic process.

Among the 17 GO processes enriched across all histotypes, several pathways central to tumorigenesis and metastasis were identified. NES values revealed distinct histotype-specific patterns: EC and HGSC showed pathway enrichment, while MC and CCC exhibited depletion (Fig. 4E). This suggests that EC and HGSC may activate pathways central to their tumorigenesis. Pathways with high NES variation across histotypes included those involved in vasculature development, cell-matrix adhesion, and regulation of peptidyl-tyrosine phosphorylation, critical processes in tumor progression and metastasis.

### Hallmark cancer pathway analysis

Using the 50 cancer-related hallmark gene sets from the Molecular Signatures Database (MSigDB), 35 pathways were significantly enriched (padj < 0.05): seven pathways shared across all histotypes, i.e., Coagulation, Complement, Epithelial-Mesenchymal Transition, Interferon Gamma Response, KRAS Signaling Up, TNFalpha Signaling via NFKB, and Xenobiotic Metabolism (Fig. 4F). These pathways showed a consistent pattern of enrichment-depletion, with (CCC/MC) depleted and (HGSC/EC) enriched (Fig. 4F). These results highlight unique regulatory mechanisms even in general oncogenic processes, indicating potential histotype-specific therapeutic targets. Additionally, distinct hallmark cancer pathway patterns were observed in three histotypes. In CCC, depletion in the Glycolysis, PI3K-AKT-MTOR signaling, and Reactive Oxygen Species pathways suggest disrupted metabolic processes. MC uniquely enriched in Spermatogenesis, and depleted in TGF-beta signaling, indicating altered growth factor responses. HGSC displayed enriched pathways related to cell regulation such as “Apical Surface, Estrogen Response, MYC Targets V2” while DNA Repair and Mitotic Spindle pathways were depleted, reflecting genomic instability and defective cell division.

### Histotype-stratified survival analysis identifies prognostic biomarker candidates in advanced-stage EOC

To identify reliable histotype-specific gene candidates associated with overall survival (OS) and disease-specific survival (DSS) in the advanced-stage EOC cohort, a stringent analytic pipeline was employed (Supplementary Data 4). Gene expression values were first z-score normalized within each histotype and dichotomized into high (z ≥ 0) and low (z < 0) expression per gene. This dichotomization of the final prognostic candidates is visualized in Supplementary Fig. 2A–D, with overall expression distributions across the cohort shown in Supplementary Fig. 2E–F. Using these z-transformed values, univariate Cox regression analysis identified between 1631 (EC) and 3974 (CCC) candidate genes associated with OS per histotype. These genes were further validated with covariate selection using LASSO, incorporating clinical parameters such as tumor stage, age at diagnosis, debulking surgery status, and initial treatment response. Multivariate Cox regression and bootstrap resampling (*p* < 0.2) identified 689 OS and 297 DSS biomarkers. After applying additional filtering to enhance robustness, the final OS biomarkers included 17 for HGSC, 119 for CCC, 133 for MC, and 302 for EC. For DSS, 13 biomarkers remained for HGSC, 115 for CCC, and 133 for MC, while none were identified for EC due to limited sample size. Biomarkers were further classified by hazard ratios (HR), with HR < 1 indicating a protective effect and HR > 1 associated with increased risk (Table 1).

For OS, favorable outcome biomarkers had C-index values ranging from 0.692 (HGSC) to 0.892 (MC), and HR values between 0.262 (EC) to 0.468 (HGSC). Unfavorable outcome biomarkers had C-index values ranging from 0.701 (HGSC) to 0.889 (MC), with HR values from 1.29 (EC) to 4.46 (EC). For DSS, C-index for favorable outcome biomarkers ranged from values 0.722 (HGSC) to 0.894 (MC), and from 0.681 (HGSC) to 0.890 (MC) for unfavorable biomarkers. HR values for favorable biomarkers ranged from 0.263 (CCC) to 0.416 (HGSC), and from 4.92 (CCC) to 3.96 (MC) for unfavorable biomarkers (Supplementary Fig. 3A-B).

**Fig. 5:**
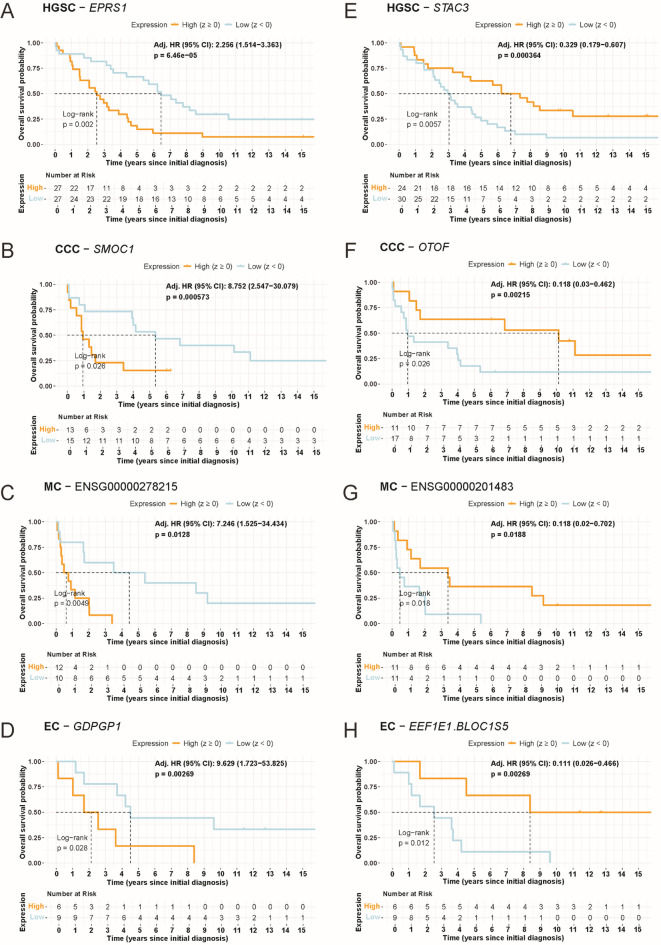
Histotype-specific prognostic genes associated with overall survival (OS). Kaplan-Meier (KM) survival curves for the top-ranked (**A-D**) unfavorable (HR > 1) and (**E-H**) favorable (HR < 1) outcome biomarkers in each histotype (HGSC, CCC, MC, and EC), based on OS. Log-rank test p-values and histotype-specific risk tables are provided for each curve, illustrating the prognostic significance of these biomarkers. Displayed adj.HR has been adjusted using the Benjamini-Hochberg model. High and low strata groups are dichotomized based on z-transformed expression data for each respective gene. HR values were adjusted by tumor stage, age at diagnosis, debulking surgery status, and initial treatment response after LASSO-selection of the covariates. Abbreviations: CCC: Clear cell ovarian carcinoma, CI: Confidence interval, DSS: Disease-specific survival, EC: Endometrioid ovarian carcinoma, HGSC: High-grade serous ovarian carcinoma, HR: Hazard ratio, MC: Mucinous ovarian carcinoma, OS: Overall survival. Note: Full DSS analyses, including additional KM plots are provided in Supplementary Fig. 3.

Kaplan-Meier (KM) plots confirmed these biomarkers by demonstrating significant survival stratification across histotypes (Fig. 5). For OS, *EPRS1* (Fig. 5A; HR: 2.26; C-index: 0.74; p-value: 0.00006) in HGSC, *SMOC1* (Fig. 5B; HR: 8.75; C-index: 0.83; p-value: 0.0006) in CCC, processed pseudogene *ENSG00000278215* (Fig. 5C; HR: 7.25; C-index: 0.92; p-value: 0.0128) in MC, and *GDPGP1* (Fig. 5D; HR: 9.63; C-index: 0.93; p-value: 0.0099) in EC were all associated with unfavorable survival outcomes representing high-risk genes. Conversely, low-risk genes included *STAC3* (Fig. 5E; HR: 0.33; C-index: 0.70; p-value: 0.0004) in HGSC, *OTOF* (Fig. 5F; HR: 0.12; C-index: 0.83; p-value: 0.0021) in CCC, ncRNA *ENSG00000201483* (Fig. 5G; *Y_RNA*; HR: 0.12; C-index: 0.91; p-value: 0.0188) in MC, as well as *EEF1E1-BLOC1S5* (Fig. 5H; HR: 0.11; C-index: 0.92; p-value: 0.0027) in EC were all linked with favorable survival outcomes.

For DSS (Supplementary Fig. 3C-H), *BARX1* (HR: 2.456; C-index: 0.69; p-value: 0.00185) and lncRNA *ENSG00000263220* (HR: 0.29; C-index: 0.75; p-value: 0.00012) in HGSC, lncRNA *ENSG00000264272* (HR: 9.61; C-index: 0.86; p-value: 0.0028) and *ENSG00000270426* (HR: 0.13; C-index: 0.90; p-value: 0.0028) in CCC, and in MC the same lncRNAs as in OS *ENSG00000278215* (HR: 7.24; C-index: 0.92; p-value: 0.0128), *ENSG00000201483* (Y_RNA; HR: 0.118; C-index: 0.91; p-value: 0.0188), and biomarkers in MC were identified as the most robust candidates. KM plots for these biomarkers demonstrated significant stratification of survival outcomes, further validating their prognostic potential. The identified OS biomarkers encompass diverse gene biotypes, including protein-coding genes (*OTOF*,* SMOC1*, and *GDPGP1*) and long non-coding RNAs (lncRNAs, *ENSG00000270426*, *ENSG00000263220*, and *ENSG00000201483* Y_RNA). This diversity reflects the complex molecular mechanisms regulating gene expression, tumor progression, and survival in EOC. In line with the discovery analysis, *STAC3* (favorable OS marker) and *BARX1* (adverse DSS marker) were validated using two RNA-based external cohorts for HGSC with the KM Plotter online tool (Supplementary Fig. 4).

## Discussion

In the present study, we performed a comprehensive analysis of advanced-stage EOC stratified by histotype reclassified according to the latest WHO classification guidelines, thereby characterizing the landscape of advanced-stage EOC histotypes^26^. Our primary objectives were to assess the impact of advances in histotype classification, identify DEGs, and define robust histotype-specific biomarker panels for diagnostic and prognostic applications. We demonstrated that nearly 47% of tumors were reassigned to new histological subtypes, highlighting the importance of continuous refinement of tumor classification. Transcriptomic profiling revealed significant genetic overlap among the HGSC, LGSC, and EC histotypes, whereas the CCC and MC histotypes exhibited distinct molecular signatures. Our DEG analysis identified histotype-specific expression patterns, leading to the construction of novel biomarker panels. Additionally, survival analysis revealed multiple histotype-specific prognostic biomarkers, an additional step towards broadening our understanding of EOC heterogeneity. Our findings provide important insights into the molecular classification of the EOC histotypes, with potential clinical applications.

This study represents the first large-scale RNA-seq-based DEG analysis of an advanced-stage EOC cohort reclassified according to the WHO 2020 guidelines. The cohort includes all major histotypes, providing a more comprehensive comparison of EOC histotypes than previous studies^27–29^. While the majority of previous studies have focused on specific histotypes or utilized single-cell RNA-seq, our approach allows for a holistic analysis of gene expression patterns, including the identification of novel biomarkers with potential diagnostic and prognostic applications^30^. Advanced-stage EOC is a major clinical challenge, as they are characterized by complex genetic heterogeneity and poor prognosis^31^. Given that up to 70% of all EOCs are diagnosed at an advanced-stage, understanding the molecular landscape of stage III/IV disease is crucial for improving clinical outcomes^32^. Frequent histotyping changes after reclassification emphasize the need for updated diagnostic frameworks in long-term cancer studies. PCA-based molecular clustering suggests that traditional histology fails to capture EOC’s genetic heterogeneity, underscoring the value of molecular classification. In breast cancer, molecular subtypes have improved prognosis and treatment selection^33,34^; applying similar strategies to EOC could enhance prognostication and personalized therapy. Established histotype-specific biomarkers such as *HNF1B*, *NAPSA*, and *WT1* were identified as DEGs, aligning with their proposed expression patterns within the respective histotypes. Although these genes were excluded from the final panels by our stringent filtering, this demonstrates the approach’s ability to identify established histotype-specific markers while elevating novel molecular candidates for EOC subgroup stratification within an advanced-stage setting.

Enrichment analysis revealed distinct pathway activation across histotypes, identifying unique biological histotype-specific pathways but also highlighting an explicit regulatory grouping among shared pathways. Overall, the identified pathways represent key tumorigenesis-related mechanisms like extracellular matrix reorganization and regulation of angiogenesis, with EC and HGSC showing enrichment, suggesting their regulation aligns with well-established cancer progression models. In contrast, CCC and MC exhibit pathway depletion, indicating that alternative molecular processes may be driving tumorigenesis in these histotypes, particularly in advanced-stage disease. These findings reinforce the importance of histotype-specific therapeutic strategies based on the unique molecular profiles of each tumor subtype. While all the genes identified in the histotype-specific gene panels provide valuable insights into EOC, we highlight the most compelling ones, particularly those with significant biological roles, strong associations with known cancer pathways, and unique genes with prognostic potential and specific to each histotype.

Analysis of HGSC revealed four key DEGs (*COL17A1*, *S100A1*, *HNF1A*, and *NPM2*), which have been implicated in critical processes such as tumor invasion, metastasis and cell signaling, tumor dedifferentiation and metabolic regulation, and chromatin instability, supporting their roles in aggressive HGSC pathogenesis and poor prognosis^35–38^. Associated with better OS in HGSC, the prognostic marker *STAC3* has previously been identified in cervical and renal clear cell cancer but has not been studied in EOC^39–41^. Conversely, *EPRS1*, linked to WNT/GSK-3β/β-catenin signaling, was found to predict poorer survival, aligning with previous research on liver cancer and ER-positive breast cancer^42,43^.

*ARIDA3A*, *ATP11A* - *ATP11A-AS1*,* C16orf74 -* lncRNA *ENSG00000287125*, and *KSR1* were prominent candidates for CCC. *ARID3A*, a transcription factor associated with the nuclear matrix, inhibits differentiation and promotes proliferation. While *ARID1A* is frequently mutated in CCC, recent studies have linked *ARID3A* mutations to CCC pathogenesis^44,45^. Despite their distinct functional subgroups, *ARID3A* and *ARID1A* may converge on key pathways critical to CCC development. The concurrent upregulation of both *ATP11A* and its antisense transcript *ATP11A-AS1*, as well as *C16orf74* and its lncRNA *ENSG00000287125*, suggests a functional coupling in transcriptional regulation, a phenomenon receiving more attention within cancer research recently^46,47^. Additionally, *KSR1*, a scaffold protein for the RAS-RAF-MEK-ERK pathway, emerged as a CCC-specific DEG, highlighting the role of Ras-driven tumor regulation in this histotype. As an established modulator of Ras, *KSR1* has been implicated in tumor progression, cell survival, and favorable prognosis across multiple cancers^48,49^ further supporting its potential significance in CCC. *OTOF*, a low-risk prognostic gene candidate for CCC is linked to Calcium-dependent membrane processes, e.g. fusion, repair, and remodeling^50^. Proposed as a prognostic biomarker for renal clear cell carcinoma, *OTOF* overexpression correlates with more unfavorable DSS contrasting its potential protective role in CCC^51^. Additionally, *SMOC1* is a calcium binding matricellular protein that is associated with epithelial cells, TGFbeta signaling, and angiogenesis^52^. Interestingly, *SMOC1* has emerged as a candidate biomarker for unfavorable clinical outcome for CCC.

For MC, key genes included *ERN2*, *CREB3L1*, *LGALS4*, and *LINC00958*. *ERN2* (IRE1 β) plays a role in epithelial homeostasis, mucin production, and ER stress regulation^53^. Previous studies have linked *ERN2* to poor prognosis for lung and pancreatic ductal cancer^54,55^. The transcription factor *CREB3L1* has been linked to metastasis and is associated with poorer survival in anaplastic thyroid carcinoma^56^. Additionally, *CREB3L1* acts as a metastatic repressor in triple-negative breast cancer and was proposed as a potential target for immunotherapy in OC via the PCNA and Wnt/β-Catenin Pathway^57–59^. *CELSR2*, a non-classic cadherin, holds prognostic value in endometrial and liver cancer^60–62^. *LGALS4*, a galactin family protein involved in tumor progression, has shown elevated expression in 66% of EOCs, with our findings now providing new insights into its distinct expression in MC^63^. *LINC00958* has been linked to cancer progression and metastasis is upregulated in several cancers, including EOC^64^. Due to the lack of existing literature, little is known about the role of the MC-specific prognostic markers, the ncRNAs *ENSG00000201483* and *ENSG00000278215*, in cancer thereby highlighting their unexplored role in cancer research and the need for further functional characterization.

Identifying a gene panel for EC was challenging due to its significant genetic overlap with HGSC. Key candidates such as *EYA1*, *SCUBE2*, and *PAX9* were prominent. *EYA1* is a transcriptional regulator linked to poor survival in advanced-stage EOC^65,66^, while *SCUBE2*, a tumor suppressor, is associated with better prognosis in multiple cancers^67–70^. Dysregulation of *PAX9* influences tumorigenesis and affects cell fate determination, with implications for therapeutic response in ovarian cancer, particularly in relation to platinum-based treatments^71,72^. The low-risk prognostic gene in EC, *EEF1E1-BLOC1S5*, is a readthrough transcript linked to global protein synthesis. While its sibling gene, *EEF1A2*, has protective effects in serous ovarian cancers, *EEF1E1* overexpression correlates with poor prognosis in liver cancer, suggesting a dual function for the *eEF1* family as both cancer-driving and protective depending on the context^73–75^. The high-risk gene *GDPGP1*, involved in neuronal glycogen regulation and stress resistance, lacks prior evidence linking it to cancer, though its speculative role in EC warrants further investigation^76^. Our integrative analysis of the protein-coding panel members revealed strong concordance between transcriptomic expression and protein abundance across histotype-specific candidates in an advanced-stage EOC, showing ATP11A (CCC), ARID3A (CCC); and LGALS4 (MC) even being “histotype-specific” on the protein level and four more genes being validated on the protein level (HGSC: COL17A1, ARSL, S100A1; CCC: KSR1). This cross-platform validation supports the biological robustness of our findings and highlights their potential utility - not only as transcript-based biomarkers but also as candidates detectable at the proteomic level. Such consistency between mRNA and protein expression underscores the translational potential of our panel, suggesting feasibility for application in both molecular diagnostics and functional follow-up studies.

While our study provides valuable insights into the transcriptomic landscape of advanced-stage EOC, several limitations must be acknowledged. As our findings are based on transcriptomic data, further validation is needed on the protein level using tissue microarrays (TMAs) and proteomic approaches, while non-coding elements require validation using quantitative real-time PCR (qRT-PCR) and RNA in situ hybridization (RNA ISH). This should be followed by functional in vivo and in vitro studies for both translated and untranslated genes. Additionally, our relatively small sample size for rare histotypes (MC, CCC, and EC) may impact statistical power, necessitating the need for larger cohorts stratified by histotype. External validation efforts were restricted due to discrepancies in histotype classification in publicly available datasets and lack of clinical data. This highlights the need for better curated, histotype-stratified multiomics resources aligned with contemporary classification frameworks. Future studies integrating multiomics data and larger sample sizes are essential to ensure the robust validation of the identified biomarkers. Furthermore, while our analysis focused on advanced-stages, future research should assess whether similar molecular patterns exist in early-stage EOCs.

In this study, we present advances in the molecular classification of advanced-stage EOC by integrating histotype reclassification with transcriptomic analysis, a significant step forward to understanding the molecular heterogeneity of advanced-stage EOC. The identification of histotype-specific biomarker panels and prognostic candidates, along with unique and shared biological processes regulating progression and tumorigenesis in each histotype, provide a foundation for personalized treatment approaches. Future research should focus on validating these biomarkers in larger cohorts, and integrating genomic, transcriptomic, and proteomic data, along with functional studies, is warranted to refine molecular classification and therapeutic approaches for EOC.

## Materials and methods

### Cohort design

The patient cohort presented in this study comprises 156 individuals diagnosed with EOC between 1993 and 2018 at Sahlgrenska University Hospital (Gothenburg, Sweden). The primary inclusion criterion was tumor staging of stage III and IV according to the International Federation of Gynecology and Obstetrics (FIGO) guidelines. Furthermore, the cohort was designed to achieve a fairly equal distribution of four of the five main histological subtypes of EOC: HGSC (29%), EC (26%), CCC (23%), and MC (23%). This selection was based on material availability and survival outcomes.

Primary invasive EOC samples were retrieved from the fresh-frozen tumor biobank at the Department of Oncology at Sahlgrenska University Hospital. Clinicopathological features were obtained from the Swedish National Cancer Registry at the National Board of Health and Welfare (Stockholm, Sweden) and the National Quality Registry at the Regional Cancer Center West (Gothenburg, Sweden). In accordance with the 2020 WHO guidelines, the selected ovarian tumors were reclassified by histotype at the Department of Clinical Pathology, Sahlgrenska University Hospital. A board-certified gynecologic pathologist conducted the reclassification using hematoxylin & eosin (H&E)-stained sections of formalin-fixed paraffin-embedded (FFPE) tissues, which were sourced from the hospital archives, prepared from diagnostic blocks, or newly generated by dehydrating and embedding fresh-frozen tumor tissue. Seven samples were excluded from the downstream analysis due to limited tissue availability or reclassification outside of EOC.

### Whole-transcriptome RNA sequencing

Total RNA was extracted from fresh-frozen tumor tissue using the RNeasy Lipid Tissue Mini Kit (Qiagen). Inclusion criteria for RNA extraction required ≥ 60% neoplastic cellularity, determined by cytological assessment of touch imprints stained with Mayer’s Grünwald and reviewed by a board-certified pathologist. Quality control of the extracted RNA was conducted using the 4200 Tapestation system (Agilent) and the Qubit RNA HS Assay Kit (ThermoFisher Scientific), resulting in measurements of RNA integrity number (RIN) and RNA concentration. The generated RIN values negatively correlated with sample age; therefore, RIN-based pre-sequencing exclusions were not applied. Whole-transcriptome RNA sequencing approach was executed at the SNP&SEQ Technology Platform via the National Genomics Infrastructure (NGI) at the Science for Life Laboratory in Uppsala, Sweden. Library preparation was performed using Illumina Stranded TotalRNA-Prep Ligation with RiboZero Plus kit. Sequencing was conducted on a NovaSeq 6000 system (S4 flow cell) generating paired-end reads of 150 bp for all samples. Comprehensive RNA quality and alignment metrics, including per-sample QC statistics and mapping efficiency, are provided in Supplementary Data 5.

### Quality control, mapping, and alignment of the Raw RNA sequencing data

High-performance computational preprocessing of the raw data was performed using SNIC SENS resources (UPPMAX project ID sens2022542). FASTQ files were trimmed to remove adapters and poly-G-tail using the bbduk tool (BBtools v.38.08; Bushnell B 2024). Quality control was subsequently performed with FastQC (v.0.11.9; Andrews, S. 2010) and the results were summarized using MultiQC (v.1.12; Ewels P et al. 2016). Trimmed reads were mapped to the human reference genome (GRCh38.p13) using the STAR aligner software (v.2.7.9a; Dobin, A. et al. 2013). Alignment quality was assessed with the MultiQC. Three samples were excluded from the DEG analysis due to bacterial contamination observed in the sample sequence reads and low overall quality scores. Quantification and count matrix generation were subsequently performed using featurecounts (subread v.2.0.0; Liao, Y et al. 2014).

### Differential gene expression analysis (DEG)

Of the initial 156 patients (including 10 samples that were excluded due to genomic contamination or the reclassification process), 146 tumor samples were included in the DEG analysis performed in R (v.4.4.0) using Deseq2 (v.1.40.1; Love, Huber et al. 2014). After normalization and variance stabilization, a filtering threshold was applied to exclude genes with less than five counts in fewer than four samples. The results of the DEG analysis were further adjusted using the apeglm shrinkage algorithm (Zhu A et al. 2018). Pairwise expression comparisons were conducted between all seven included subgroups (HGSC, CCC, MC, EC, LGSC, borderline tumors (BOT), and benign tissue), with results filtered for statistical significance based on a Benjamin-Hochberg adjusted p-value < 0.05 and log2FoldChange (log2FC) > 1.0. Histotype-specific gene panels for the four main histotypes (HGSC, CCC, MC, EC; herein presented in this order) were generated to extract DEGs that showed differential expression unique to each histotype compared to all others.

### DEG validation procedure

The resulting ‘histotype-specific’ DEGs underwent internal validation through a two-step approach. First, logistic regression models were trained for each DEG using repeated 10-fold cross-validation with the caret package (v6.0–93). Model performance was evaluated based on ROC AUC, sensitivity, and specificity using the pROC package (v1.18.0). Second, a random forest model was trained on all DEGs to evaluate combined predictive power using the randomForest package (v4.7-1.1.1). A global Importance Score was assigned to each tested gene, enabling ranking and the selection of top candidates for each histotype. Annotated DEGs with Hugo Gene Nomenclature Committee (HGNC) symbols were externally validated using four RNA expression datasets (GSE2109^77^, GSE6008^78,79^, GSE44104^80^, E-MTAB-1814^81^; retrieved from the Gene Expression Omnibus (GEO, https://www.ncbi.nlm.nih.gov/geo/) or ArrayExpress (https://www.ebi.ac.uk/biostudies/arrayexpress). Supplementary Table 3 summarizes the cohort characteristics of the external datasets, with data curation details provided in^82^. The aim of this external validation was testing the consistency of expression patterns for the original study-cohort DEGs across the four main histotypes. Therefore, DEG analysis for these external cohorts was performed using the Limma package (v.3.60.2), with FDR correction (padj < 0.05). To ensure robustness, only consistent DEGs found across multiple external datasets were considered (*Total Frequency* refers to the overall proportion of datasets in which the gene was identified as differentially expressed. Consistency was defined as a matching direction of effect (log2 fold change) between the external datasets and our DEG analysis. This was quantified via *Relative Frequency*, representing the proportion of relevant comparisons (with the respective histotype as reference) in which the gene was differentially expressed. A threshold of ≥ 33% for *Relative Frequency* was set for a gene to be considered externally validated, and this flag was appended to our protein-coding gene panel biomarker candidates (Supplementary Data 6 for external DEG statistics of the validated DEG’s for HGSC, CCC, and MC).

### Proteomic expressional analysis in advanced-stage EOC

An in-house advanced-stage cohort with proteomics data was used to validate the expression patterns of histotype-specific candidates. The cohort is nearly paired with the EOC subset of this study cohort (118 of 119 samples are paired between transcriptomics and proteomics) and is comprised of the four main histotypes (HGSC, CCC, EC, and MC). Proteomic sample preparation and raw data processing were described elsewhere^83^. Data access is available via MassIVE repository (MSV000097588).

Differential abundance analysis was performed with the NormalyzerDE package (v. 1.20.0) in R using limma two-group contrasts (unpaired). For each protein-coding gene candidate and histotype, the proportion of available histotype contrasts where the protein expression patterns were in the same direction as RNA expression (e.g., UP in RNA and UP in protein expression) and statistically significant (adjP < 0.05; |log2FC| ≥ 1.5) was assessed. Three tiers were considered for further reporting: strict (100% of contrasts significant and concordant; histotype-specific DAP), externally validated (≥ 67% of contrasts), and a direction-only tier (same direction in ≥ 67% of contrasts, ignoring significance).

### Gene set enrichment analysis (GSEA)

Gene set enrichment analysis was performed on histotype-specific DEGs, comparing the histotype of interest against all other samples grouped as “others”. Genes were ranked by log2FoldChange values. GSEA was conducted using the gseGO function from the clusterProfiler package (v.4.12.6; Yu, G., et al., 2012) with Gene Ontology Biological Process (BP) terms applying a significance threshold of p-value < 0.05. To complement GSEA, enrichment analysis was also performed using the fgsea algorithm (v1.30.0; Korotkevich, G., et al., 2021) with pathway data obtained from the msigdbr package (v.7.5.1; Dolgalev, I., 2022). Additional tools included biomaRt (v.2.58.2; Durinck, S., et al., 2005, 2009) for gene ID conversion and the org.Hs.eg.db package (v3.19.1) for gene annotations.

### Survival analysis

Overall survival was defined from diagnosis to death from any cause, and disease-specific survival from diagnosis to death due to EOC, with cause of death classified using the National Cause of Death Register. If the date of death was unavailable or the patient was lost to follow-up, data were censored on March 11, 2024, as the administrative cutoff. OS and DSS analyses were performed using a structured analysis pipeline. Initially, histotype-specific expression matrices (DESeq2’s VST) were filtered, followed by z-score normalization within each histotype to dichotomize data into high (z ≥ 0) and low (z < 0) expression groups, corresponding to above- and below-average expression per gene, which were then used as input for subsequent survival analyses. Genes showing significant associations (*p* < 0.05) in these univariate analyses were further tested using Kaplan-Meier analysis to assess potential overfitting. Genes that were significant in both the OS and DSS analyses underwent covariate testing using Lasso regularization. Clinical covariates included tumor stage, age at diagnosis, debulking surgery status, and initial treatment response. Multivariate Cox regression models were subsequently fitted to the data, followed by permutation-based bootstrapping with 1,000 iterations to assess the robustness and calculate empirical p-values (*p* ≤ 0.2). Additional filtering excluded overfitted hits (concordance index [C-index] ≠ 1; confidence interval span ≠ 1; hazard ratio [HR] outside the range of 0.1 to 10) resulting in robust prognostic biomarker candidates. The analysis pipeline utilized the following R packages: survival (v3.7-0) for survival modeling, RegParallel (v1.22.0) for parallelized regression, glmnet (v4-1.8) for Lasso regulation, and boot (v1.3–30) for bootstrapping. Kaplan-Meier plots were generated using the survminer package (v0.4.9). The prognostic biomarker candidates for HGSC (*BARX1* and *STAC3*) were further validated using the KM Plotter web-based tool with RNA microarray (GEO accession ID GSE14764) and RNA-seq data (TCGA) for ovarian cancer^84,85^. The following settings were selected in KM Plotter: (1) Auto select best cutoff (percentile) to stratify the patient cohort, (2) clinical endpoint (OS, RFS [recurrence-free survival] or PFS [progression-free survival]), and (3) only ‘Jetset’ best probe set (for RNA microarray only).

## Supplementary Information

Below is the link to the electronic supplementary material.

Supplementary Material 1

Supplementary Material 2

Supplementary Material 3

Supplementary Material 4

Supplementary Material 5

Supplementary Material 6

Supplementary Material 7

Supplementary Material 8

Supplementary Material 9

Supplementary Material 10

Supplementary Material 11

## Data Availability

The RNA-seq data, presented in this study, have been deposited in the NCBI Gene Expression Omnibus (http://www.ncbi.nlm.nih.gov/geo/) under accession number GSE295399. All additional data are available under their corresponding Gene Expression Omnibus or ArrayExpress accession codes.
