# Supplementary material for: Diagnostic and prognostic biomarkers associated with histotype in advanced epithelial ovarian cancer
Source: Sci Rep. 2025 Oct 23;15:37171. doi: 10.1038/s41598-025-24938-0 (PMC12550092; doi:10.1038/s41598-025-24938-0)
Supplement: Supplementary file 1 — Supplementary Material 1 [file 41598_2025_24938_MOESM1_ESM.docx]

**Supplementary Information**

**Manuscript Title:**Diagnostic and prognostic biomarkers associated with histotype in advanced epithelial ovarian cancer

**Authors:**Ella Ittner^1,2^, Hugo Swenson^1,2^, Lucas Werner^1,2^, Elisabeth Werner Rönnerman^1,2,3^, Constantina Mateoiu^3^, Anikó Kovács^3^, Pernilla Dahm-Kähler^4^, Ghassan Saed^5,6,7^, Per Karlsson^1,8^, Toshima Z. Parris^1,2^, and Khalil Helou^1,2^

**Affiliation:**^1^Department of Oncology, Institute of Clinical Sciences, Sahlgrenska Academy, University of Gothenburg, Gothenburg, Sweden, ^2^Sahlgrenska Center for Cancer Research, Sahlgrenska Academy, University of Gothenburg, Gothenburg, Sweden**,** ^3^Region Västra Götaland, Sahlgrenska University Hospital, Department of Clinical Pathology, Gothenburg, Sweden**,** ^4^Department of Obstetrics and Gynecology, Institute of Clinical Sciences, Sahlgrenska Academy, University of Gothenburg, Gothenburg, Sweden**,** ^5^Department of Obstetrics and Gynecology, Wayne State University School of Medicine, Detroit, MI, USA**,** ^6^Department of Gynecologic Oncology, Karmanos Cancer Institute, Detroit, MI, USA**,** ^7^Department of Obstetrics and Gynecology, University of Jordan School of Medicine, Amman, Jordan**,** ^8^Region Västra Götaland, Sahlgrenska University Hospital, Department of Oncology, Gothenburg, Sweden

**Corresponding Author:** Ella Ittner; email: Ella.ittner@gu.se

This file includes:

**Supplementary Table 1.** Full cohort characteristics (n = 156), including all malignant, borderline, benign, and excluded cases, stratified by histotype following WHO 2020 reclassification.

**Supplementary Table 2.** Cohort characteristics describing the analytic EOC subset. Covering all included samples of HGSC, CCC, MC, and EC histotypes reclassified according to WHO 2020.

**Supplementary Table 3:** External dataset characteristics, including histotype representation and platform details, for the four RNA expression datasets (GSE2109, GSE6008, GSE44104, EMTAB1814) used for external validation procedure for histotype-specific DEG’s.

**Supplementary Figure 1.** Cumulative incidence plot covering the four main histotypes (HGSC, CCC, EC, MC), stratified by histotype and cause of death (death from EOC vs. other causes).

**Supplementary Figure 2.** Expression of prognostic biomarker candidates. Z-score-based discotimazed boxplots and vst-based expression plots

**Supplementary Figure 3.** Concordance index (C-index) distribution for histotype-specific overall survival (OS) and disease-specific survival (DSS) models, and prognostic genes associated with DSS in each histotype.

**Supplementary Figure 4.**

**Supplementary Data Sets (submitted as separate Excel files):**

- **Supplementary Data 1:** Differential gene expression results across histotype comparisons (apeglm, DESeq2)
- **Supplementary Data 2:** Gene set enrichment analysis results (GO and MSigDB Hallmark)
- **Supplementary Data 3:** Histotype-specific gene panels with validation metrics
- **Supplementary Data 4:** Prognostic survival model results (OS and DSS)
- **Supplementary Data 5:** RNA quality control (QC), alignment metrics and relevant sample characteristics for the full cohort (156 samples) and the final study cohort (146 samples)
- **Supplementary Data 6:** DEG and histotype-specific DEG panels results from external validation procedure (using (GSE2109, GSE6008, GSE44104, E-MTAB-1814)
- **Supplementary Data 7**: Differential abundance results across histotype comparisons (nearly paired proteomics cohort, NormalyzerDE output) and summary table comparing directionality and histotype-representation between DEG’s and DAP’s

**Supplementary Table 1** Clinicopathological characteristics of the full, reclassified advanced-stage EOC cohort (n = 156), stratified by histotype according to WHO 2020 guidelines. This table includes all seven cohort subgroups: five malignant epithelial ovarian cancer (EOC) histotypes—HGSC, CCC, EC, MC, and LGSC—as well as borderline tumors (BOT), benign cases, and samples excluded from downstream analysis due to reclassification.

Abbreviations: BOT: borderline tumor; CCC: clear cell carcinoma; EC: endometrioid carcinoma; HGSC: high-grade serous carcinoma; LGSC: low-grade serous carcinoma; MC: mucinous carcinoma; PCA: principal component analysis; WHO: World Health Organization.

**Supplementary Table 2** Clinicopathological characteristics of the analytic EOC subset stratified by histotype. This cohort includes malignant, advanced-stage primary tumors from four histotypes—HGSC, CCC, EC, and MC—reclassified according to WHO 2020 criteria and used for downstream transcriptomic analysis, including biomarker profiling, functional enrichment, and prognostic evaluation. Full cohort characteristics are provided in Supplementary Table 1. Abbreviations: CCC, clear cell carcinoma; EC, endometrioid carcinoma; EOC, epithelial ovarian cancer; HGSC, high-grade serous carcinoma; MC, mucinous carcinoma; WHO, World Health Organization.

**Supplementary Table 3** Cohort characteristics of the four external data sets used for external validation: GEO series GSE2109, GSE6008, GSE44104, and the ArrayExpress dataset E-MTAB-1814 analysis. The table summarizes the number of samples per histotype, publication year, platform, and data type for each dataset. Abbreviations: CCC, clear cell carcinoma; EC, endometrioid carcinoma; GEO: gene expression omnibus; GSE: gene expression omnibus series; HGSC, high-grade serous carcinoma; MC, mucinous carcinoma.

**Supplementary Figures**

**
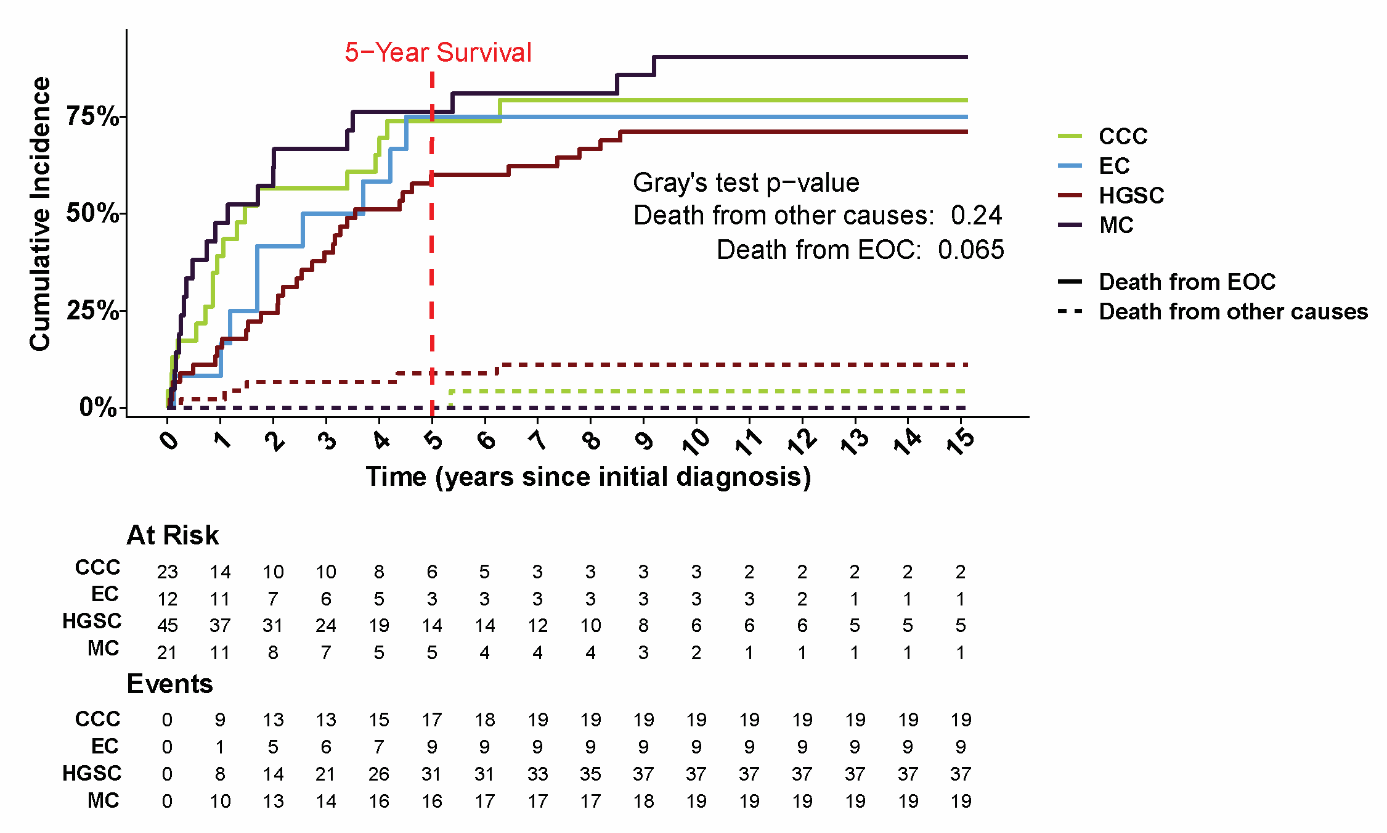
**

**Supplementary Fig. 1 Cumulative survival probabilities across the four major EOC histotypes.** Curves depict the cumulative incidence of death from EOC within the advanced-stage cohort, stratified by histotype (HGSC, CCC, MC, EC). Deaths from other causes were treated as competing events. Gray’s test performed to assess differences in cause-specific mortality across histotypes. Time is shown in years since initial diagnosis, with a 15-year follow-up cut-off; 5-year survival is highlighted with a dotted line. Abbreviations: HGSC, high-grade serous ovarian carcinoma; CCC, clear cell ovarian carcinoma; MC, mucinous ovarian carcinoma; EC, endometrioid ovarian carcinoma; EOC, epithelial ovarian cancer.

**
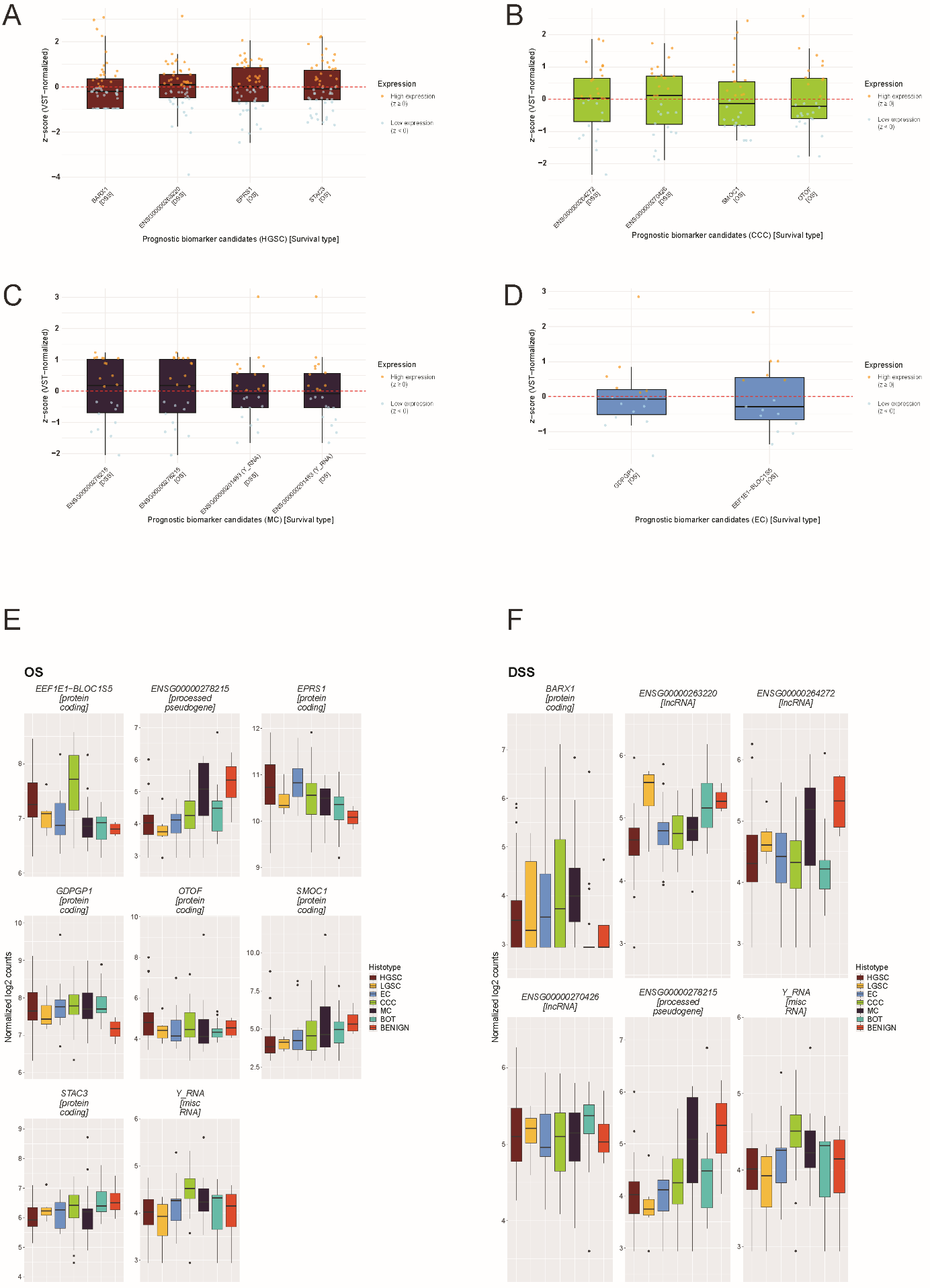
**

**Supplementary Fig. 2** **Expression patterns of histotype-specific prognostic biomarker candidates in advanced-stage EOC.** Boxplots (A-D) show the expression distributions of each prognostic biomarker candidate across samples (histotype-specific), stratified by high (z ≥ 0) and low (z < 0) expression groups based on z-score normalization following DESeq2’s VST, with z = 0 representing the average expression within the respective histotype. A red dotted line is included to indicate this threshold. These boxplots illustrate the dichotomization used in the survival analysis for OS and DSS. E and F provide the variance-stabilized expression profiles for each biomarker, illustrating the full expression variability across the cohort for the same prognostic biomarker candidates. Abbreviations: CCC: Clear cell ovarian carcinoma, DSS: Disease-specific survival, EC: Endometrioid ovarian carcinoma, EOC: epithelial ovarian cancer; HGSC: High-grade serous ovarian carcinoma, MC: Mucinous ovarian carcinoma, OS: Overall survival, VST: Variance stabilization transformation.

**
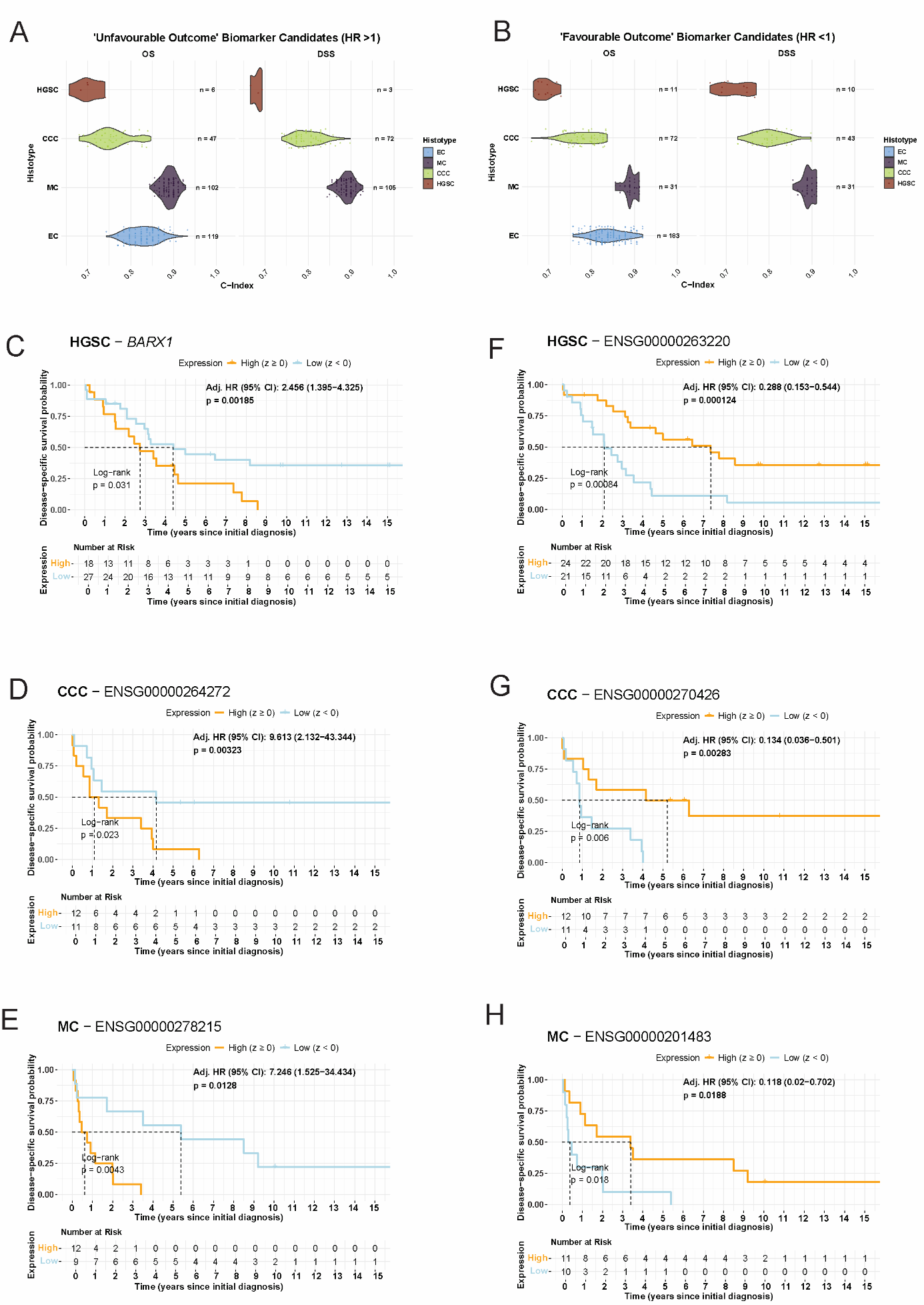
**

**Supplementary Fig. 3** C-Index Distribution and Histotype-specific with disease specific survival associated prognostic genes. (A-B) Distribution of C-index values stratified by histotype, showing biomarkers associated with (A) favorable outcomes (HR < 1) and (B) unfavorable outcomes (HR > 1)); (C-H) Kaplan-Meier (KM) survival curves for the top-ranked (C-E) favorable (HR < 1) and (F-H) unfavorable (HR > 1) outcome biomarkers in the histotypes HGSC, CCC, and MC based on disease specific survival (DSS). Log-rank test p-values and histotype-specific risk tables are provided for each curve, illustrating the prognostic significance of these biomarkers. Displayed adj.HR has been adjusted using the Benjamini-Hochberg model. High and low strata groups are dichotomized based on z-transformed expression data for each respective gene. HR shown were adjusted by tumor stage, age at diagnosis, debulking surgery status, and initial treatment response after LASSO-selection of the covariates. Abbreviations: CCC: Clear cell ovarian carcinoma, CI: Confidence interval, DSS: Disease-specific survival, EC: Endometrioid ovarian carcinoma, HGSC: High-grade serous ovarian carcinoma, HR: Hazard ratio, MC: Mucinous ovarian carcinoma.

**
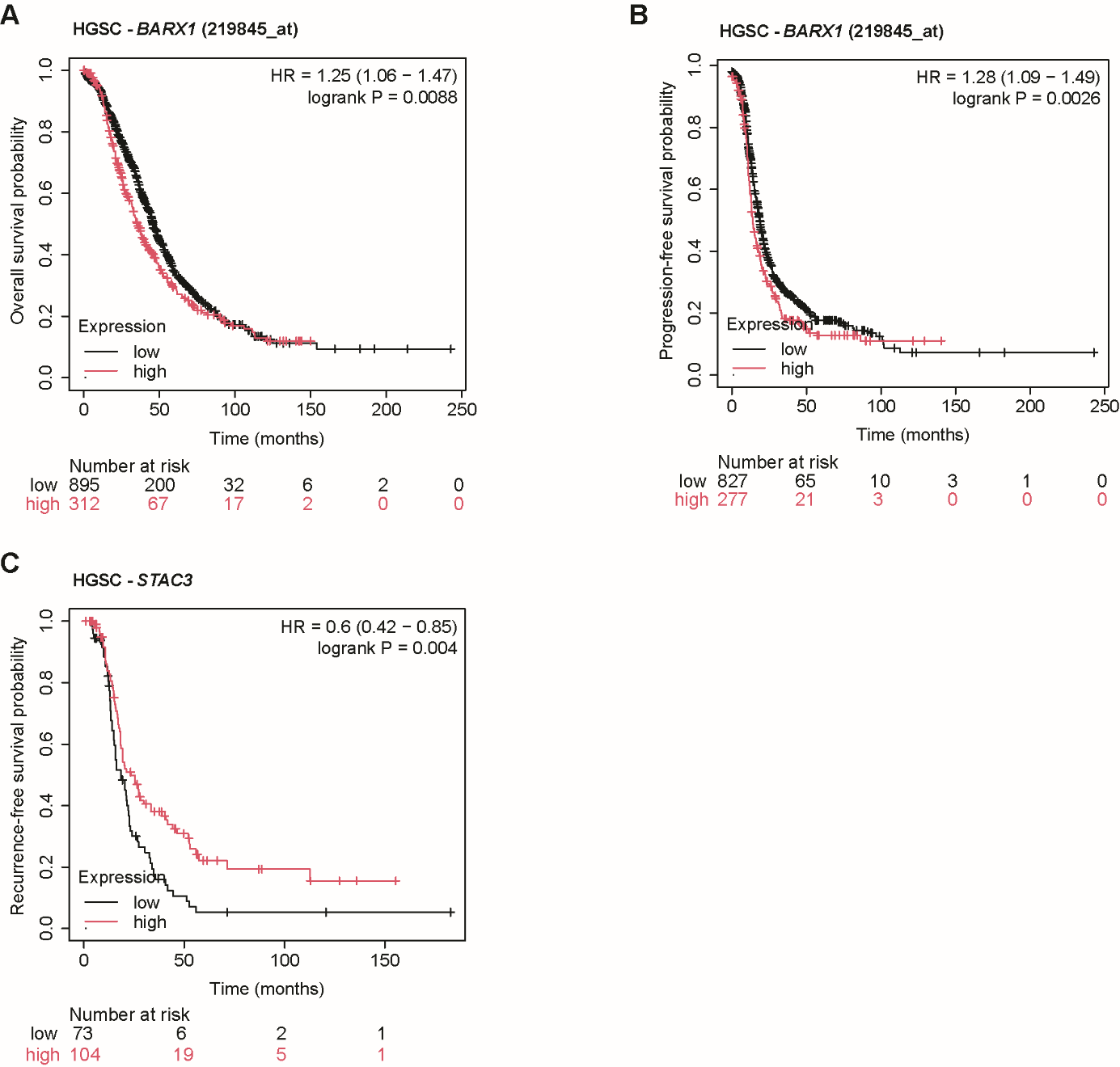
**

**Supplementary Fig. 4** Kaplan-Meier curves from the KM plotter web-based tool showing the association between (A) BARX1 expression and overall survival (OS) for ovarian cancer using the GSE14764 dataset, (B) BARX1 expression and progression-free survival (PFS) for ovarian cancer using the GSE14764 dataset, and (C) STAC3 expression and recurrence-free survival (RFS) for ovarian cancer using the TCGA-OV dataset. High BARX1 expression was associated with significantly more unfavorable OS and PFS for ovarian cancer, while low STAC3 expression was associated with unfavorable RFS.
